# Effects of different intensities of physical exercise on insulin sensitivity and protein kinase B/Akt activity in skeletal muscle of obese mice

**DOI:** 10.1590/S1679-45082014AO2881

**Published:** 2014

**Authors:** Rodolfo Marinho, Leandro Pereira de Moura, Bárbara de Almeida Rodrigues, Luciana Santos Souza Pauli, Adelino Sanchez Ramos da Silva, Eloize Cristina Chiarreotto Ropelle, Claudio Teodoro de Souza, Dennys Esper Corrêa Cintra, Eduardo Rochete Ropelle, José Rodrigo Pauli

**Affiliations:** 1 Universidade Estadual Paulista Júlio de Mesquita Filho, Rio Claro, SP, Brazil; 2 Universidade Estadual de Campinas, Limeira, SP, Brazil; 3 Universidade de São Paulo, Ribeirão Preto, SP, Brazil; 4 Universidade do Extremo Sul Catarinense, Criciúma, SC, Brazil

**Keywords:** Physical exercise, Obesity, Diabetes, Insulin resistance, Mice

## Abstract

**Objective:**

To investigate the effects of different intensities of acute exercise on insulin sensitivity and protein kinase B/Akt activity in skeletal muscle of obese mice.

**Methods:**

Swiss mice were randomly divided into four groups, and fed either a standard diet (control group) or high fat diet (obese sedentary group and obese exercise group 1 and 2) for 12 weeks. Two different exercise protocols were used: swimming for 1 hour with or without an overload of 5% body weight. The insulin tolerance test was performed to estimate whole-body sensitivity. Western blot technique was used to determine protein levels of protein kinase B/Akt and phosphorylation by protein Kinase B/Akt in mice skeletal muscle.

**Results:**

A single bout of exercise inhibited the high fat diet-induced insulin resistance. There was increase in phosphorylation by protein kinase B/Akt serine, improve in insulin signaling and reduce of fasting glucose in mice that swam for 1 hour without overload and mice that swan for 1 hour with overload of 5%. However, no significant differences were seen between exercised groups.

**Conclusion:**

Regardless of intensity, aerobic exercise was able to improve insulin sensitivity and phosphorylation by protein kinase B/Ak, and proved to be a good form of treatment and prevention of type 2 diabetes.

## RESUMO

**Objetivo:**

Investigar os efeitos do exercício físico agudo com diferentes intensidades sobre a sensibilidade à insulina e a atividade da proteína quinase B/Akt no músculo esquelético de camundongos obesos.

**Métodos:**

Foram utilizados camundongos *Swiss*, divididos aleatoriamente em quatro grupos, que receberam dieta padrão (grupo controle) ou dieta hiperlipídica (grupos obeso sedentário e grupos obesos exercitados 1 e 2), por período de 12 semanas. Dois diferentes protocolos de exercício foram utilizados: natação durante 1 hora com ou sem sobrecarga de 5% da massa corporal. O teste de tolerância à insulina foi realizado para estimar a sensibilidade à insulina. E os níveis protéicos da proteína quinase B/Akt e de sua fosforilação foram determinados no músculo esquelético dos camundongos, através da técnica de *Western blot*.

**Resultados:**

Uma sessão de exercício físico foi capaz de inibir a resistência à insulina em decorrência de uma dieta hiperlipídica. Foi possível demonstrar um aumento na fosforilação da proteína quinase B/Akt, melhora da sinalização da insulina e redução da glicemia de jejum nos camundongos que realizaram 1 hora de natação sem sobrecarga adicional e nos camundongos que realizaram 1 hora de natação com sobrecarga adicional de 5% de sua massa corporal. Entretanto, não houve diferença significativa entre os grupos que realizaram o exercício em diferentes intensidades.

**Conclusão:**

Independente da intensidade, o exercício físico aeróbio conseguiu aumentar a sensibilidade à insulina e a fosforilação da proteína quinase B/Akt, revelando ser uma boa forma de tratamento e prevenção do diabetes tipo 2.

## INTRODUCTION

Physical exercise increases insulin sensitivity independently of body mass reduction and changes in body composition.^(1)^ The major effect of exercise is the increase in intracellular proteins expression of insulin signaling pathway especially the glucose transport in skeletal muscle.^(1-3)^

Obese individuals and resistant to insulin have as common characteristics the problem in insulin signaling due to subclinical inflammatory process. This process shows a decrease in action of this hormone in target cells and is influenced by the presence of fasting hyperglycemia and hyperinsulinemia. Such metabolic changes are associated with a decrease in glucose uptake by skeletal muscle in response to insulin.^(4)^

Among mechanisms involved in insulin signaling, the distal protein by molecular pathway of this hormone, *i.e.*, the serine/threonine protein kinase B/Akt, is emphasized. Protein Akt, widely studied in the last years, is an important physiological regulator that controls several cellular functions such as growth, survival, proliferation and metabolism.^(5)^

Protein Akt has three isoforms (Akt1, Akt2 and Akt3), and each isoform exerts a specific function inside the cell^(5)^. A previous study showed that Akt1 knockout mice have small body size and increased apoptosis rates than their wild type mice. That situation revealed the importance of this isoform for cellular survival.^(6)^ Akt2 knockout mice developed characteristics of type 2 *diabetes mellitus* that were crucial for maintenance of glycemia. Animals with protein Akt3 absence had an impairment in brain development.^(5-8)^

Intracellular insulin signaling begin with its linking with a specific receptor of the membrane, a heterotetrameric protein with kinase activity, composed by two alpha subunits and two beta subunits named insulin receptor (IR).^(9)^ The IR activation results in phosphorylation on tyrosine of several substrates, including insulin receptor substrates 1 and 2 (IRS-1 and IRS-2). After phosphorylation on tyrosine the IRS-1 and IRS-2 can bind and activate phosphatidylinositol 3-kinase (PI3-kinase). This protein triggers phosphorylation of membrane phosphoinositides and the recruiting of Akt close to the plasmatic membrane. The phosphoinositides promote and activate phosphorylation of phosphoinositidedependent kinase (PDK). Finally, PDK activates the Akt when phosphorylating it in its catalytic domain (Thr^308^). The activated Akt protein has the ability to phosphorylate and activate several metabolic targets. The Akt, for example*,* stimulates the glucose uptake, synthesis of glycogenic and proteins in skeletal muscle.^(5,10-12)^

The glucose uptake stimulated by Akt in skeletal muscle involves phosphorylation and activation of Akt substrate, which is a protein of molecular weight of 160 kDa, named AS160. When phosphorylated in threonine 642 this protein is dissociated from vesicles that contain glucose transporters type 4 (GLUT-4), enables the translocation and exocytosis of these vesicles into the membrane, and increases GLUT-4 expression which consequently increases glucose uptake.^(10,12)^

Currently physical exercise is considered one of the most important strategies for prevention and treatment of insulin resistance and type 2 *diabetes mellitus*. Studies on cells *in vitro*, experimental animal model of diabetes and with human patients showed that a single bout of exercise increased the insulin sensibility. This effect was associated with higher phosphorylation (activation) of Akt and its substrates in skeletal muscle. As a consequence, we observed an increase of expression and translocation of GLUT-4 transporter and glucose uptake in skeletal muscle during and after physical exercise.^(13-17)^

It is clear in the literature that physical exercise promotes significant improvement on insulin signaling in skeletal muscle and on insulin sensibility, however, further investigation should be done to identify physiological and molecular effects of physical exercises of different intensities in organisms with insulin resistance and diabetes.

## OBJECTIVE

This study investigated the effects of different intensities of acute exercise on insulin sensibility and Akt phosphorylation in skeletal muscle of obese mice.

## METHODS

### Characterization of animals

All experiments followed care principles and procedures for the use of experimental animals and were approved by ethical committee of the *Universidade Estadual de Campinas* (UNICAMP), protocol nº 2805-1. The experiments were conducted in the first semester 2013. The sample included 24, 6-weeks-old, Swiss mice bred at the Multidisciplinary Center for Biological Research at UNICAMP. Animals were housed in single cages and had free access to water and two types of diet: standard rodent chow (C) or hyper fat diet (HFD) during experimental period. Details of diets are given in table 1. Mice were exposed to 12 hour light/dark cycles and temperature of 20°C - 22°C. Animals were randomly divided into four subgroups: control group (n=6) fed with standard chow (C); sedentary obese group (n=6) fed with HFD for 12 weeks (OB); obese group (n=6) fed with HFD for 12 weeks and submitted to acute exercise without overload (OE-1); and obese group (n=6) that also received HFD for 12 weeks and were submitted to acute exercise with overload of 5% of animals' body weight (OE-2).

**Table 1:** Composition of standard rodent chow and hyper fat diet

Ingredients	Standard rodent chow		Hyper fat diet	
	g/kg	Kcal/kg	g/kg	Kcal/kg
Corn starch (e.q.t.)	398	1590	116	462
Casein	200	800	200	800
Sucrose	100	400	100	400
Dextrinized starch	132	528	132	528
Pork fat	-	-	312	2808
Soybean oil	70	630	40	360
Cellulose	50	-	50	-
Mineral mixture	35	-	35	-
Vitamin mixture	10	-	10	-
L-Cystine	3	-	3	-
Choline	2.5	-	2.5	-
Total	1000	3948	1000	5358

g/kg: gram/kilogram; Kcal/kg: calorie/kilogram; e.q.t.: enough quantity to.

### Protocol for acute exercise

The acute exercise protocol consisted in animals swimming, where animals swan in group of six at a cylindrical tank of 45 cm in length and 60cm depth, with water temperature of 32± 1°C. Animals in group OE-1, exercised in single bout for 1 hour without additional overload, and animals in group OE-2 exercised for the same time with overload of 5% of body weight attached to animals' tail. Before the beginning of bout of exercises, to acclimatize to water, animals were placed in the swimming tank with water up to chest level for three consecutive days. The other groups of animals were submitted to the same procedure to simulate the stress in the water received by group OE-1 and OE-2. All experiments were conducted 24 hours after the exercise bout and with previous fasting for 8 hours.

### Assessment of metabolic parameters

At the end of experimental period, animals total body mass (digital balance), glucose and insulin blood levels were assessed. In addition, animals of each experimental group were submitted to insulin tolerance test (ITT).

### Determination of glucose and insulin

Plasmatic glucose dosage was conducted using the enzymatic colorimetric of glucose oxidase. Plasmatic insulin samples were evaluated by the ELISA method.

### Insulin tolerance test

ITT was conducted 24 hours after the bout of swimming exercise. Animals had been fasting for 8 hours before blood sample was collection to verify the glycemia at time 0 of the test. Subsequently, insulin (2U/Kg of body mass) was injected intraperitoneally and then blood samples were collected from the animals' tail at 5, 10, 15, 20, 25 and 30 minutes to determine the blood glucose value using a portable blood glucose meter (Advantage, Boehringer, Mannheim, Germany). Glucose decay constant rate (Kitt) was measured using the formula 0.693/t_1/2_. The glucose t_1/2_ was measure from analysis curve of minimum squares of glucose concentration during the linear decay phase.^(18)^

### Extraction of skeletal muscles and fat tissue

Mice were anesthetized using intraperitoneal injection of ketamin/diazepam (50/5mg/kg). The level of anesthesia was monitored by the corneal and pedal reflexes. Animals' abdominal cavity was opened and after the portal vein was identified, 0.2mL of saline solution or insulin (10^-6^ mol/l) was injected. We collected samples of gastrocnemius muscles (mixed portion) after 90 seconds of insulin that were homogenized in immunoprecipitation buffer containing 1% Triton X 100M Tris (pH 7.4), 100mM of sodium pyrophosphates, 100mM of sodium fluoride, 10mM of EDTA, 10mM of sodium vanadate, 2mM of PMSF and 0.1mg/mL of aprotinin at 4°C. The homogenized was centrifuged at 11,000 rpm for 30 minutes. The protein concentration was determined in supernatant solution by Bradford method.^(19)^ Subsequently, a total extract determination was conducted and an immunoprecipitation assay with specific antibodies. At the end of the experimental procedures, the epididymal adipose tissue was removed for assessment of its total mass using an analytical balance.

#### Western blot

After determine proteins concentration the western blot technique was applied and specific antibodies were used. The samples were separated, after a rapid boiling, by polyacrylamide gel electrophoresis (SDS-PAGE). Separated proteins in SDS-PAGE were transferred to nitrocellulose in BIO-RAD media transfer device. Nitrocellulose membrane was incubated overnight using a specific antibody. The linking of antibody to non-specific proteins was minimized by pre-incubation of nitrocellulose membrane with blocking buffer (5% of powdered fat free milk; 10mmol/L Tris; 150mmol/L of NaCI; 0.02% Tween 20) for 1.5 hour. The identifying method used was the chemiluminescence in which proteins of interest are detected by incubation of membranes with primary antibodies and subsequently with conjugated-secondary antibodies with horseradish peroxidase (HRP). Immunoreactive bands were detected by the chemiluminescence and densitometry determined by uptake system and imaging analysis.

### Antibodies

Antibodies used for immunoblot experiment were anti-Akt and anti-phosphoserine Akt (Ser473) (Santa Cruz Biotechnology, CA, EUA).

### Statistical analysis

Results were expressed as mean±standard error of the mean. The Student's *t* test was used for non-paired data to compare the two groups. When necessary the variance analysis was used with the Bonferroni post hoc test for multiple comparisons of means. The significance level of p<0.05 was adopted.

## RESULTS

### Physiologic and metabolic parameters

Table 2 compares data of mice control groups (C), OB, OE-1 and OE-2. We observed that total body mass of animals that received high fat diet was significantly higher than those animals from the control group. However, there was no difference among groups (OB, OE-1 and OE-2). Similar results could be observed for weight of total epididymal fat. However, no differences were seen among groups of obese animals induced by diet.

**Table 2:** Physiologic and metabolic parameters

	Body mass (g)	Epididymal fat (g)	Fasting glycemia (mg/dL)	Fasting insulin (ng/ml)
C (n=6)	28.4±1.88	0.6±0.10	98.6±3.96	2.9±0.62
OB (n=6)	51.4±3.12*	2.9±0.16*	306±26.60*	8.8±0.62*
OE-1 (n=6)	53.6±2.88*	3.1±0.12*	186±21.40**	9.0±0.62*
OE-2 (n=6)	56.2±3.33*	3.1±0.21*	154±31.50**	8.9±0.62*

* p<0.05: C *versus* OB, OE-1 and OE-2;

** p<0.05: OB *versus* OE-1 and OE-2.

C: control group; OB: sedentary obese group; OE-1: obese group that exercised without overload; OE-2: obese group that exercised with overload.

Fasting glycemia values found in animals of groups OB, OE-1 and OE-2 were significantly higher than the control group. Mice that exercised from OE-1 and OE-2 had satisfactorily higher hexose than the group OB. But no statistical difference was found among groups submitted to difference intensities of acute exercise.

Insulinemia values were significantly higher in mice of group OB, OE-1 and OE-3 than in the control group. This result states that acute exercise did not change insulin values in animals.

### Phosphorylation of protein Akt in skeletal muscle

**Figure 1A-B:**
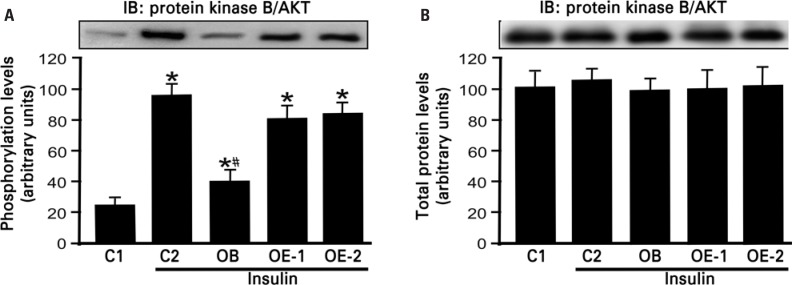
Phosphorylation and protein kinase B/Akt expression in skeletal muscle (gastrocnemius). C1: control with saline injection; C2: control with insulin injection; OB: obese group with insulin injection; OE-1: exercise obese with insulin injection without overload and OE-2: exercised obese group with insulin injection and with overload. IB: immunoblotting technique. *p<0.05, different of C1; ^#^p<0.05: OB different of C2, OE-1 and OE-2

We verified that phosphorylation of Akt in skeletal muscle increased in all experimental groups (C2, OB, OE-1 and OE-2) when stimulated by insulin compared to control group that received saline (C1) (Figure 1A). In OB mice, phosphorylation of Akt was reduced 2.3 times after insulin injection when compared with mice in the control group (C2). In animals of group OE-1 and OE-2 the phosphorylation of Akt increased 2.0 and 2.1 times, respectively, compared with animals of group OB (Figure 1A). No statistical difference was seen in phosphorylation of Akt between groups OE-1 and OE-2. The Akt expression did not show statistical difference among studied groups (Figure 1B).

### Acute exercise increases sensibility to insulin

**Figure 2:**
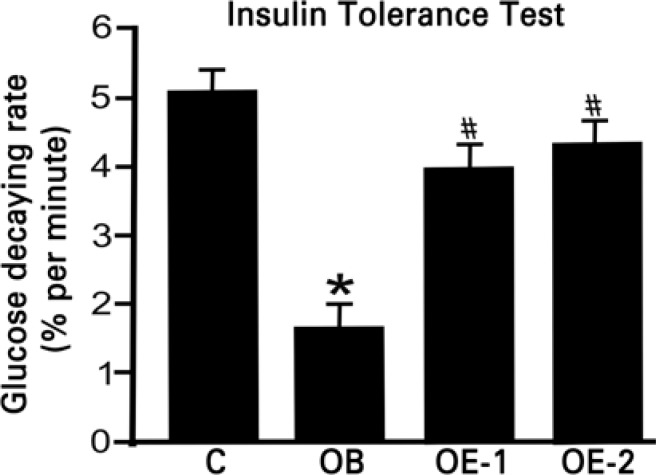
Insulin tolerance test. C: control group; OB: obese group; OE-1: exercised obese group without overload and OE-2: exercised obese group with overload. *p<0.05, different of C, ^#^p<0.05, different of OB

We verified that sedentary mice fed with high fat diet had lower rate of glucose uptake than mice in the control group (C: 5.0±0.49; OB: 1.78±0.68 %/min). However, mice that performed acute exercise had an increase in glucose uptake rate in TTI (OE-1: 3.98±0.45; OE-2: 4.33±0.72) compared with sedentary obese animals, but these values did not differ from the control group. Such results showed that a single bout of exercise can increase glucose uptake in obese mice (Figure 2).

## DISCUSSION

Studies have shown that insulin sensibility increases with practice of acute or chronic physical exercise, even when there is no reduction of body fat mass.^(1,3,20)^ This study did not find difference in total body mass and epididymal fat content between experimental groups. This result was expected once a single bout of exercise is not enough to produce changes in body composition of mice. However, the protocol for acute swimming exercise was able to increase insulin sensibility and phosphorylation of Akt in obese animals. This protein constitutes a key for insulin signaling pathway.

Luciano et al.^(21)^ verified that sensibility and insulin signaling (by IRS/PI3-kinase/Akt) in skeletal muscle and epydidymal fat tissue of lean mice increased after six weeks of swimming training with attached overload corresponding to 5% of animal body mass. This fact showed that exercise is efficient to increase the insulin response in peripheral tissues sensitive to insulin, such as skeletal muscle and fat of healthy animals.

However, in obesity there is an increase in production and release of pro-inflammatory cytokines, from the fat tissue infiltrated with macrophages, along with increase of free fatty acids from lipolysis and intake of saturated fat-rich diet that cause damages to insulin signaling and hyperglycemia.^(22)^ Recent studies had observed that some types of fatty acids from diet, especially the saturated fatty acids, are capable to linking and activating proteins of membrane named TollLikeReceptor 4 (TLR4). When activated, these receptors trigger an inflammatory response and this activation promotes negative consequences for insulin signaling pathway.^(3,22)^ This occurs because TLR4 has the ability to active proteins serine-kinase IKKβ (Ikappakinase beta) and JNK (c-jun N-terminal kinase), known as pro-inflammatory action. In this sense, these proteins promote phosphorylation in serine 307 of insulin receptor and insulin receptor subtracts 1 and 2 (IRS1 and 2) decreasing the insulin signal in different tissues.^(3,22)^

In addition, the TLR4 receptor triggers activation of IKK/I
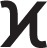
B/NF
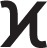
B pathway. When this protein is activated, the IKKβ promotes phosphorylation of I
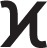
B that provokes dissociation of I
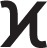
B/NF
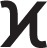
B complex. Because it is a transcription factor, when it free inside the cell, the NF
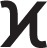
B translocate to the nucleus and promotes transcription of new inflammatory cytokines, such as tumor necrosis factor alpha (TNF-α), protein phosphatases as the protein phosphatases 1B (PTP1B), and inducible nitric oxide synthase (iNOS) with recognized negative effect on molecular insulin signal. For this reason, an increase of inflammatory subclinical feature is associated with obesity and consequently with the insulin resistance state in obesity.^(3,22)^

In this inflammatory feature associated with obesity and insulin resistance, researchers of several parts of the world have been investigating the role of physical exercise to restore insulin sensibility, reduce activating of TLR4, serine-kinase proteins JNK and IKKβ in obese animals.^(3)^ Oliveira et al.^(3)^ observed that suppression of inflammatory pathway lasted for 24 and 36 hours after the last bout of exercise. These authors used a protocol of swimming exercise for rodents, the similar model used in our study. However, Oliveira et al sample was composed by Wistar rats that, although develop obesity and insulin resistance, do not always present hyperglycemia as Swiss mice.

These findings are consistent with Ropelle et al.^(20)^ who observed increase of sensibility to insulin in obese rats submitted to acute exercise protocol. Although, these author's study used a protocol with high volume of exercises with bouts for 3 hours each and intervals of 45 minutes. Besides that, no additional overload was used during swimming exercise so that these exercises could be considered of low intensity.

Da Silva et al.^(23)^ compared two physical exercise protocols of different volume and intensity and observed that the protocol of high volume of exercise (6 hours with additional overload) and the protocol of low volume of exercise (45 minutes with weight of 5.5% of animal's body mass [equivalent to maximum lactate steady state]) were efficient to restore insulin sensibility, suppress protein expression and phosphorylation of serine kinase, JNK and IKKβ, and increase protein phosphorylation of insulin signaling pathway (IR, IRS-1 and Akt). Therefore, these authors showed that shortterm exercise can promote similar results of those found with long-term exercises. Although these results are of great importance to the literature because low volume of exercise is more easily tolerable by obese, especially for the difficult caused by obesity in performing of exercises that require body mass movement. Unfortunately, these authors did not check differences in responses of different intensity exercises at low volume protocols.

In our study, the obese Swiss mice induced by high fat diet were insulin resistant and had low phosphorylation of Akt protein than lean animals from the control group. A different result was seen in animals submitted to acute exercise protocol for 1 hour, without or with additional weight of 5% of body mass, that were more sensible to insulin and had an increase in phosphorylation of Akt than obese mice that not exercised. However, independently of physical exercise protocol these variables did not present difference. This result indicates that obese animals that exercised without additional weight attached to their body had similar benefit as those that exercised with more intensity (performed with additional weight). These results have a great relevance for practical application of physical exercise protocols in obese population considering that low intensity and volume aerobic exercises are more easily tolerable by this public, mainly in the beginning of practice of exercise.

O'Gorman et al. when studied the increase of induced glucose uptake by exercise in obese individuals with type 2 diabetes reported that an acute bout of exercise for 1 hour to 75% of VO_2peak_, done in stationary bicycle, was enough to increase sensibility to insulin in those individuals.^(24)^ However, after seven bouts of exercise with the same volume and intensity of acute bout of exercise, researchers observed increases in glucose uptake by the hyperinsulinemic-euglycemic clamp test and expression of type 4 glucose transporter (GLUT4) in obese patients with type 2 diabetes. However, when they analyzed proteins of insulin signaling they did not observe differences between basal status and after physical exercise.^(24)^ This phenomenon could be explained because exercise can stimulate independent pathways of insulin to increase glucose uptake as the pathway of the kinase protein activated by AMP (AMPK).^(10,16)^ The AMPK is activated in situations of energetic deficit and increased GLUT4 translocation for muscle cell membrane. In addition, because physical exercise can achieve glucose uptake in other insulinsensitive tissues, such as fat tissue.^(25)^

In our study we observed that acute exercise could increase insulin sensibility in obese animals. This change occurred at least in parts for the increase of Akt protein phosphorylation in skeletal muscle of exercised obese rodents. However, exercise has shown to be able to increase glucose uptake in response to insulin also in fat tissue. Peres et al.^(25)^ submitted animals to physical training for seven weeks and they observed higher response of signaling pathway of insulin in adipocytes with increasing in glucose uptake and phosphorylation in tyrosine of insulin receptor subtracts 1 and 2 (IRS-1 and IRS-2), high association of IRS-1 with PI3-Kinase and, as a consequence, increase Akt protein phosphorylation. Besides, physical exercise, as previous reported, stimulates glucose uptake by AMPK pathway favoring the entrance of these hexoses in contracted muscle,^(16,17)^ even when the signaling pathway of insulin is impaired.

Although in our study did not evaluate this aspects, it is possible that they collaborate for the increase of total body insulin sensibility observed insulin tolerance test in obese animals submitted to acute exercise and, for this reason, which had contributed for homeostasis of glucose in these animals. These mechanism needs to be further investigate in different physical exercise protocols.

Other study composed by obese individuals and obese with type 2 diabetes by Christ-Roberts et al.^(26)^ reported that eight weeks of aerobic exercises for 45 minutes and 70% VO_2peak_ of intensity were efficient to increase glucose uptake in both obese volunteers and obese with diabetes. Physical training also enabled an increase in GLUT4 expression of Akt protein in the two groups, however, this increase in Akt expression was not followed by increase in phosphorylation neither by changes in other proteins of insulin signaling pathway. Hence, the importance of further studies with acute and chronic exercise protocols in obese and obese with diabetes population is clear, particularly to understand better the effects of physical exercise on insulin signaling pathway.

In addition, the increase of Akt phosphorylation with exercise could be a result of an improvement in inflammatory process observe by previous studies in the literature.^(3,22,23)^ As previously presented, some pro-inflammatory proteins with increased activity in obesity are able to impair the insulin signaling pathway, acting especially on proximal proteins of insulin signaling pathway. Among these proteins, the kinase serine JNK and IKK could phosphorylate in serine 307 the receptor and insulin receptor subtracts (IR and IRSs, respectively) and impair the insulin signal, which results in low Akt phosphorylation. It is well know that exercise reduces the activity of these proteins and restores insulin action.^(3,22,23)^

The most recently discovery, the TRB3 protein (a mammalian homolog of Drosophila Tribbles) has its expression increased in obesity and it has the ability to physically bind to Akt, therefore avoiding Akt activation and consequently impairing the biologic effects of this activity such as the GLUT4 translocation to the muscle cell membrane.^(27,28)^ A study conducted by Matos et al.^(28)^ showed that physical exercise could decrease TRB3 expression and restore the Akt phosphorylation in skeletal muscle in obese animals.^(28)^ This is another molecular mechanism that physical exercise can increase Akt phosphorylation. Although our study did not analyze the TRB3 expression, there is the possibility that both exercises protocols had reduced TRB3 expression and, contributed with increase in Akt phosphorylation after an acute bout of physical exercise.

## CONCLUSION

Our findings showed that physical exercise protocols of low intensity and moderate intensity are different strategies to restore insulin sensibility efficiently in obese organisms.
